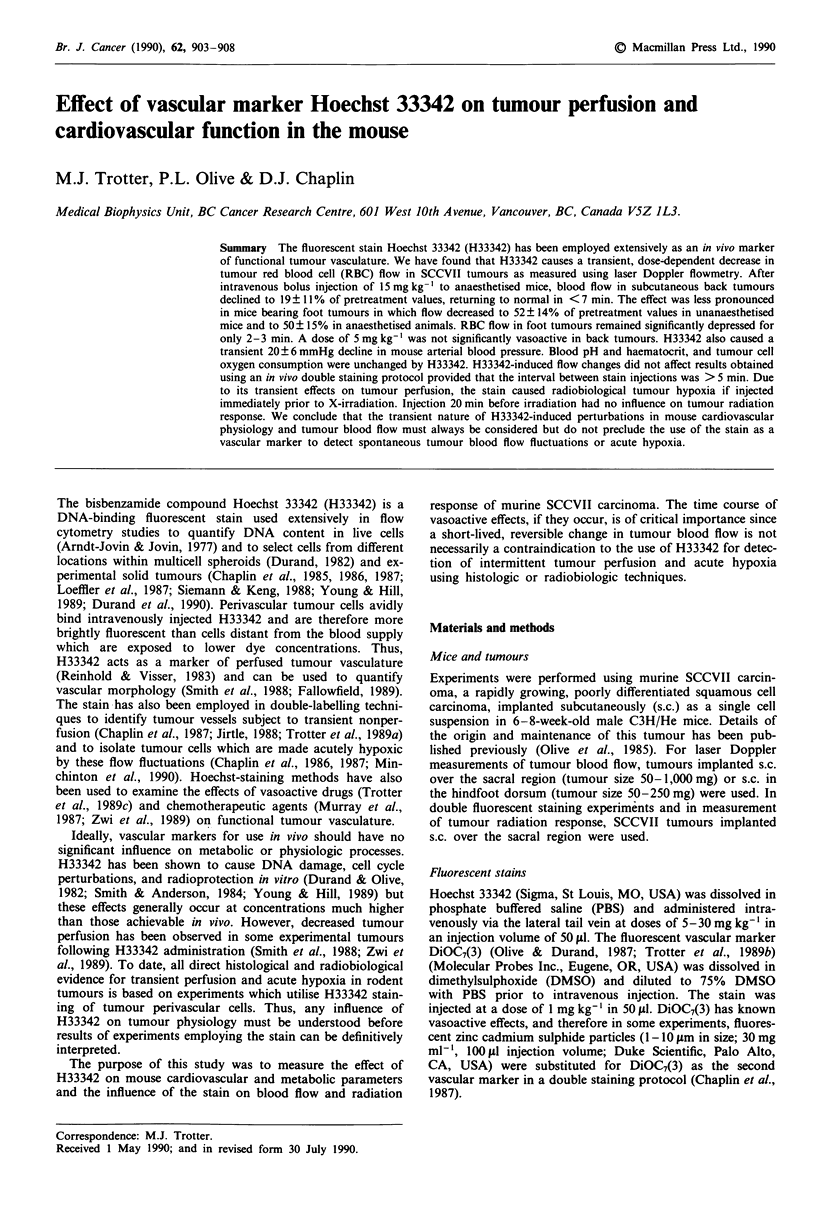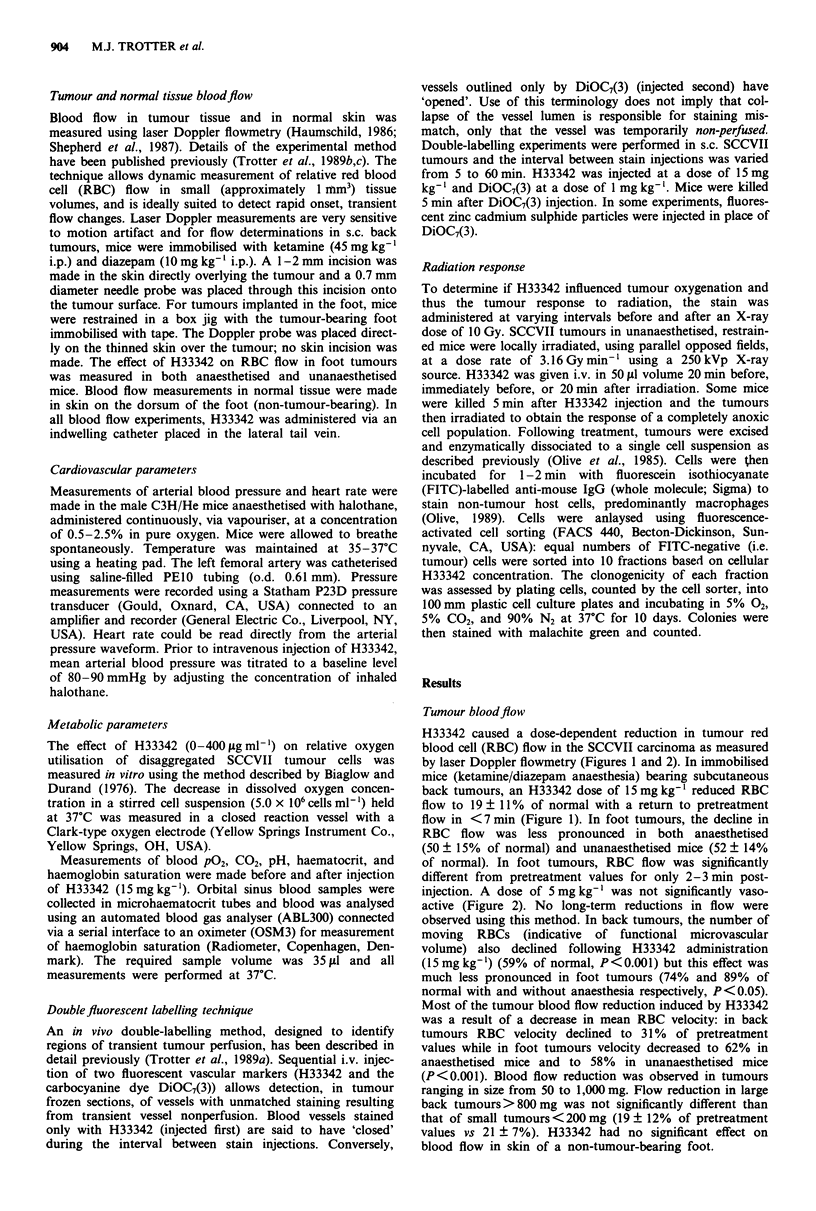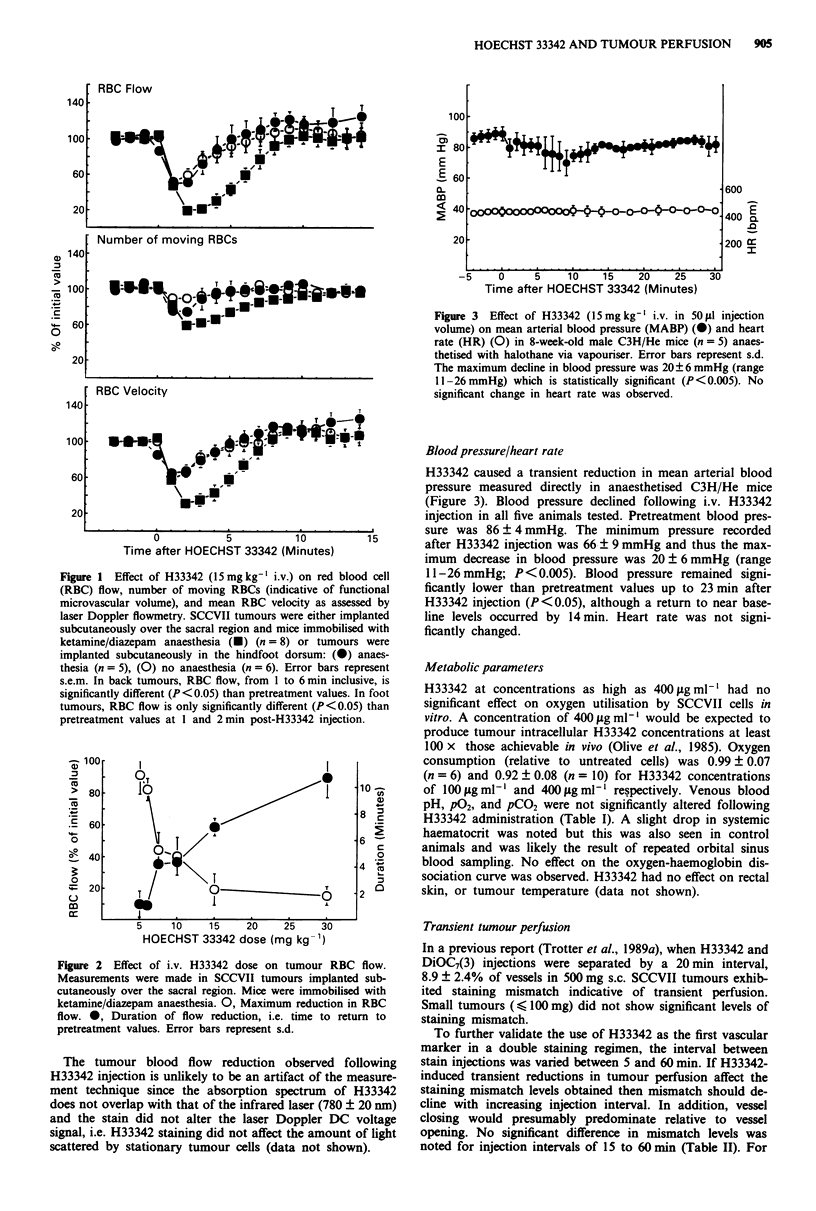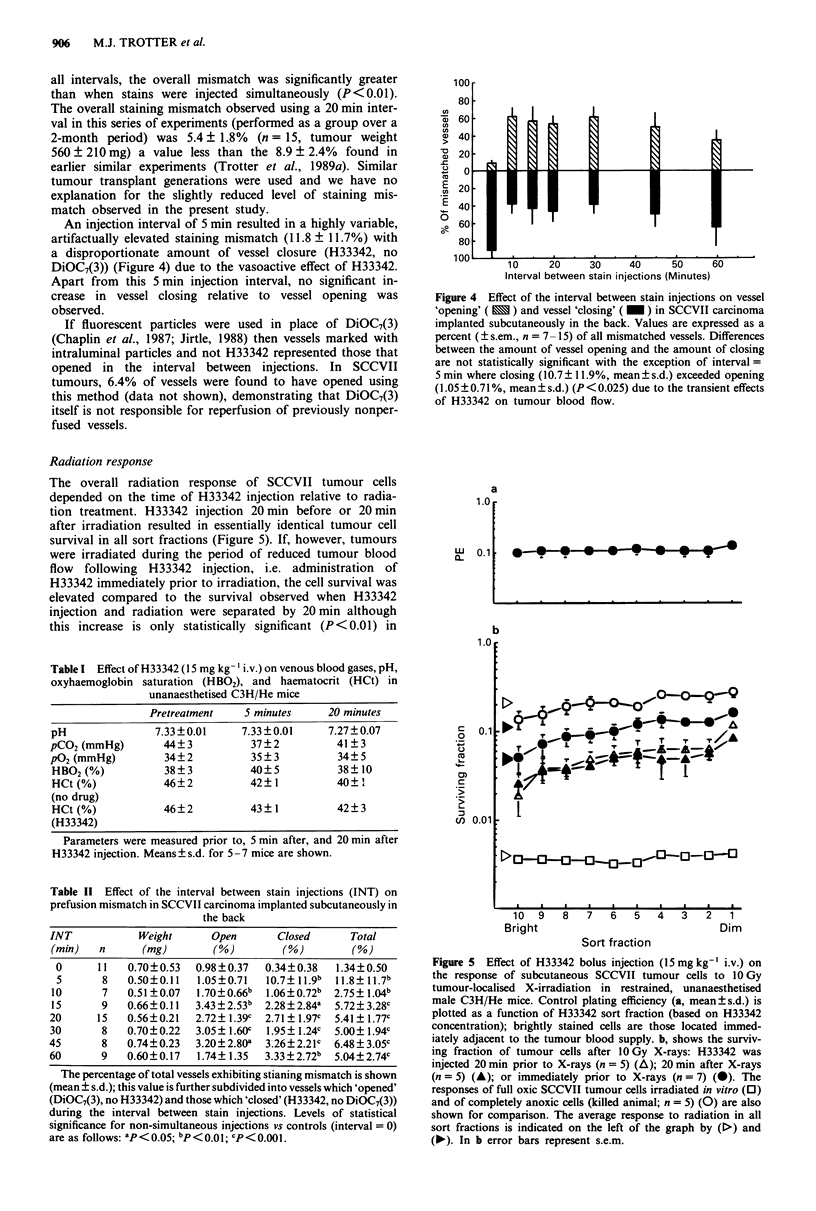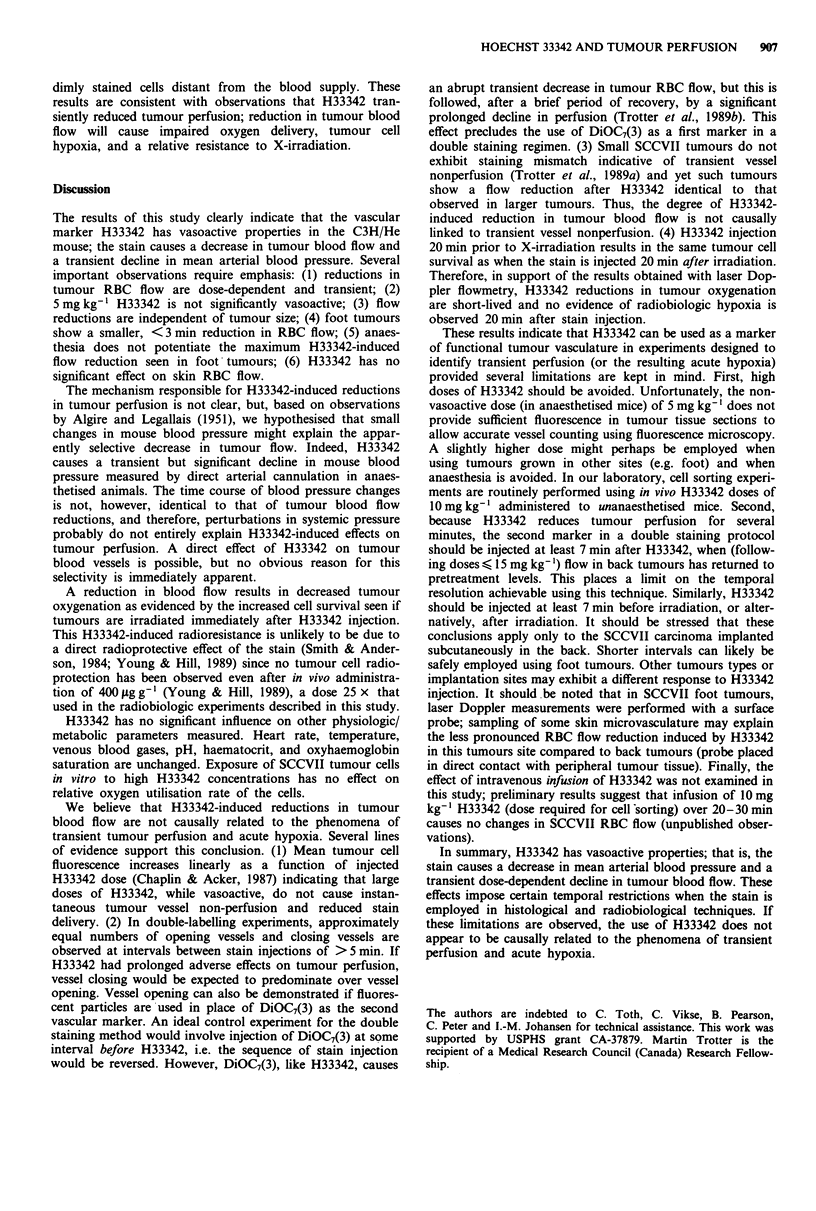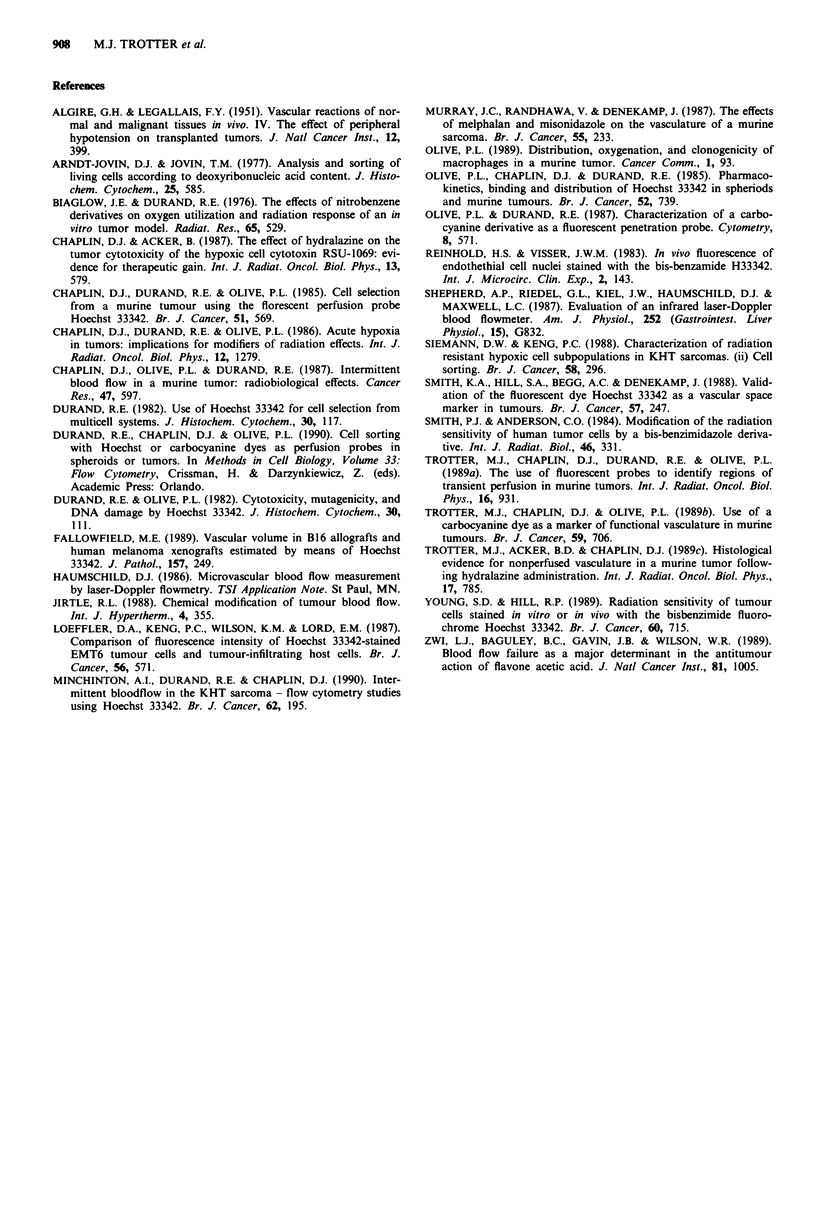# Effect of vascular marker Hoechst 33342 on tumour perfusion and cardiovascular function in the mouse.

**DOI:** 10.1038/bjc.1990.406

**Published:** 1990-12

**Authors:** M. J. Trotter, P. L. Olive, D. J. Chaplin

**Affiliations:** Medical Biophysics Unit, BC Cancer Research Centre, Vancouver, Canada.

## Abstract

The fluorescent stain Hoechst 33342 (H33342) has been employed extensively as an in vivo marker of functional tumour vasculature. We have found that H33342 causes a transient, dose-dependent decrease in tumour red blood cell (RBC) flow in SCCVII tumours as measured using laser Doppler flowmetry. After intravenous bolus injection of 15 mg kg-1 to anaesthetised mice, blood flow in subcutaneous back tumours declined to 19 +/- 11% of pretreatment values, returning to normal in less than 7 min. The effect was less pronounced in mice bearing foot tumours in which flow decreased to 52 +/- 14% of pretreatment values in unanaesthetised mice and to 50 +/- 15% in anaesthetised animals. RBC flow in foot tumours remained significantly depressed for only 2-3 min. A dose of 5 mg kg-1 was not significantly vasoactive in back tumours. H33342 also caused a transient 20 +/- 6 mmHg decline in mouse arterial blood pressure. Blood pH and haematocrit, and tumour cell oxygen consumption were unchanged by H33342. H33342-induced flow changes did not affect results obtained using an in vivo double staining protocol provided that the interval between stain injections was greater than 5 min. Due to its transient effects on tumour perfusion, the stain caused radiobiological tumour hypoxia if injected immediately prior to X-irradiation. Injection 20 min before irradiation had no influence on tumour radiation response. We conclude that the transient nature of H33342-induced perturbations in mouse cardiovascular physiology and tumour blood flow must always be considered but do not preclude the use of the stain as a vascular marker to detect spontaneous tumour blood flow fluctuations or acute hypoxia.


Br. J. Cancer (1990), 62, 903-908 © Macmillan Press Ltd., 1990

### Effect of vascular marker Hoechst 33342 on tumour perfusion and cardiovascular function in the mouse

M.J. Trotter, P.L. Olive & D.J. Chaplin

*Medical Biophysics Unit, BC Cancer Research Centre, 601 West 10th Avenue, Vancouver, BC, Canada V5Z 1L3.*

**Summary** The fluorescent stain Hoechst 33342 (H33342) has been employed extensively as an *in vivo* marker of functional tumour vasculature. We have found that H33342 causes a transient, dose-dependent decrease in tumour red blood cell (RBC) flow in SCCVII tumours as measured using laser Doppler flowmetry. After intravenous bolus injection of 15 mg $kg^{-1}$ to anaesthetised mice, blood flow in subcutaneous back tumours declined to 19±11% of pretreatment values, returning to normal in <7 min. The effect was less pronounced in mice bearing foot tumours in which flow decreased to 52±14% of pretreatment values in unanaesthetised mice and to 50±15% in anaesthetised animals. RBC flow in foot tumours remained significantly depressed for only 2-3 min. A dose of 5 mg $kg^{-1}$ was not significantly vasoactive in back tumours. H33342 also caused a transient 20±6 mmHg decline in mouse arterial blood pressure. Blood pH and haematocrit, and tumour cell oxygen consumption were unchanged by H33342. H33342-induced flow changes did not affect results obtained using an *in vivo* double staining protocol provided that the interval between stain injections was >5 min. Due to its transient effects on tumour perfusion, the stain caused radiobiological tumour hypoxia if injected immediately prior to X-irradiation. Injection 20 min before irradiation had no influence on tumour radiation response. We conclude that the transient nature of H33342-induced perturbations in mouse cardiovascular physiology and tumour blood flow must always be considered but do not preclude the use of the stain as a vascular marker to detect spontaneous tumour blood flow fluctuations or acute hypoxia.

The bisbenzamide compound Hoechst 33342 (H33342) is a DNA-binding fluorescent stain used extensively in flow cytometry studies to quantify DNA content in live cells (Arndt-Jovin & Jovin, 1977) and to select cells from different locations within multicell spheroids (Durand, 1982) and experimental solid tumours (Chaplin *et al.*, 1985, 1986, 1987; Loeffler *et al.*, 1987; Siemann & Keng, 1988; Young & Hill, 1989; Durand *et al.*, 1990). Perivascular tumour cells avidly bind intravenously injected H33342 and are therefore more brightly fluorescent than cells distant from the blood supply which are exposed to lower dye concentrations. Thus, H33342 acts as a marker of perfused tumour vasculature (Reinhold & Visser, 1983) and can be used to quantify vascular morphology (Smith *et al.*, 1988; Fallowfield, 1989). The stain has also been employed in double-labelling techniques to identify tumour vessels subject to transient nonperfusion (Chaplin *et al.*, 1987; Jirtle, 1988; Trotter *et al.*, 1989*a*) and to isolate tumour cells which are made acutely hypoxic by these flow fluctuations (Chaplin *et al.*, 1986, 1987; Minchinton *et al.*, 1990). Hoechst-staining methods have also been used to examine the effects of vasoactive drugs (Trotter *et al.*, 1989*c*) and chemotherapeutic agents (Murray *et al.*, 1987; Zwi *et al.*, 1989) on functional tumour vasculature.

Ideally, vascular markers for use *in vivo* should have no significant influence on metabolic or physiologic processes. H33342 has been shown to cause DNA damage, cell cycle perturbations, and radioprotection *in vitro* (Durand & Olive, 1982; Smith & Anderson, 1984; Young & Hill, 1989) but these effects generally occur at concentrations much higher than those achievable *in vivo*. However, decreased tumour perfusion has been observed in some experimental tumours following H33342 administration (Smith *et al.*, 1988; Zwi *et al.*, 1989). To date, all direct histological and radiobiological evidence for transient perfusion and acute hypoxia in rodent tumours is based on experiments which utilise H33342 staining of tumour perivascular cells. Thus, any influence of H33342 on tumour physiology must be understood before results of experiments employing the stain can be definitively interpreted.

The purpose of this study was to measure the effect of H33342 on mouse cardiovascular and metabolic parameters and the influence of the stain on blood flow and radiation response of murine SCCVII carcinoma. The time course of vasoactive effects, if they occur, is of critical importance since a short-lived, reversible change in tumour blood flow is not necessarily a contraindication to the use of H33342 for detection of intermittent tumour perfusion and acute hypoxia using histologic or radiobiologic techniques.

### Materials and methods

### *Mice and tumours*

Experiments were performed using murine SCCVII carcinoma, a rapidly growing, poorly differentiated squamous cell carcinoma, implanted subcutaneously (s.c.) as a single cell suspension in 6-8-week-old male C3H/He mice. Details of the origin and maintenance of this tumour has been published previously (Olive *et al.*, 1985). For laser Doppler measurements of tumour blood flow, tumours implanted s.c. over the sacral region (tumour size 50-1,000 mg) or s.c. in the hindfoot dorsum (tumour size 50-250 mg) were used. In double fluorescent staining experiments and in measurement of tumour radiation response, SCCVII tumours implanted s.c. over the sacral region were used.

### *Fluorescent stains*

Hoechst 33342 (Sigma, St Louis, MO, USA) was dissolved in phosphate buffered saline (PBS) and administered intravenously via the lateral tail vein at doses of 5-30 mg $kg^{-1}$ in an injection volume of 50 µl. The fluorescent vascular marker $DiOC_7(3)$ (Olive & Durand, 1987; Trotter *et al.*, 1989*b*) (Molecular Probes Inc., Eugene, OR, USA) was dissolved in dimethylsulphoxide (DMSO) and diluted to 75% DMSO with PBS prior to intravenous injection. The stain was injected at a dose of 1 mg $kg^{-1}$ in 50 µl. $DiOC_7(3)$ has known vasoactive effects, and therefore in some experiments, fluorescent zinc cadmium sulphide particles (1-10 µm in size; 30 mg $ml^{-1}$, 100 µl injection volume; Duke Scientific, Palo Alto, CA, USA) were substituted for $DiOC_7(3)$ as the second vascular marker in a double staining protocol (Chaplin *et al.*, 1987).

Correspondence: M.J. Trotter.
Received 1 May 1990; and in revised form 30 July 1990.

*Tumour and normal tissue blood flow*

Blood flow in tumour tissue and in normal skin was measured using laser Doppler flowmetry (Haumschild, 1986; Shepherd *et al.*, 1987). Details of the experimental method have been published previously (Trotter *et al.*, 1989*b*,*c*). The technique allows dynamic measurement of relative red blood cell (RBC) flow in small (approximately 1 $mm^3$) tissue volumes, and is ideally suited to detect rapid onset, transient flow changes. Laser Doppler measurements are very sensitive to motion artifact and for flow determinations in s.c. back tumours, mice were immobilised with ketamine (45 mg $kg^{-1}$ i.p.) and diazepam (10 mg $kg^{-1}$ i.p.). A 1–2 mm incision was made in the skin directly overlying the tumour and a 0.7 mm diameter needle probe was placed through this incision onto the tumour surface. For tumours implanted in the foot, mice were restrained in a box jig with the tumour-bearing foot immobilised with tape. The Doppler probe was placed directly on the thinned skin over the tumour; no skin incision was made. The effect of H33342 on RBC flow in foot tumours was measured in both anaesthetised and unanaesthetised mice. Blood flow measurements in normal tissue were made in skin on the dorsum of the foot (non-tumour-bearing). In all blood flow experiments, H33342 was administered via an indwelling catheter placed in the lateral tail vein.

*Cardiovascular parameters*

Measurements of arterial blood pressure and heart rate were made in the male C3H/He mice anaesthetised with halothane, administered continuously, via vapouriser, at a concentration of 0.5–2.5% in pure oxygen. Mice were allowed to breathe spontaneously. Temperature was maintained at 35–37°C using a heating pad. The left femoral artery was catheterised using saline-filled PE10 tubing (o.d. 0.61 mm). Pressure measurements were recorded using a Statham P23D pressure transducer (Gould, Oxnard, CA, USA) connected to an amplifier and recorder (General Electric Co., Liverpool, NY, USA). Heart rate could be read directly from the arterial pressure waveform. Prior to intravenous injection of H33342, mean arterial blood pressure was titrated to a baseline level of 80–90 mmHg by adjusting the concentration of inhaled halothane.

*Metabolic parameters*

The effect of H33342 (0–400 $\mu g\ ml^{-1}$) on relative oxygen utilisation of disaggregated SCCVII tumour cells was measured *in vitro* using the method described by Biaglow and Durand (1976). The decrease in dissolved oxygen concentration in a stirred cell suspension ($5.0 \times 10^6$ cells $ml^{-1}$) held at 37°C was measured in a closed reaction vessel with a Clark-type oxygen electrode (Yellow Springs Instrument Co., Yellow Springs, OH, USA).

Measurements of blood $pO_2$, $CO_2$, pH, haematocrit, and haemoglobin saturation were made before and after injection of H33342 (15 mg $kg^{-1}$). Orbital sinus blood samples were collected in microhaematocrit tubes and blood was analysed using an automated blood gas analyser (ABL300) connected via a serial interface to an oximeter (OSM3) for measurement of haemoglobin saturation (Radiometer, Copenhagen, Denmark). The required sample volume was 35 µl and all measurements were performed at 37°C.

*Double fluorescent labelling technique*

An *in vivo* double-labelling method, designed to identify regions of transient tumour perfusion, has been described in detail previously (Trotter *et al.*, 1989*a*). Sequential i.v. injection of two fluorescent vascular markers (H33342 and the carbocyanine dye $DiOC_7(3)$) allows detection, in tumour frozen sections, of vessels with unmatched staining resulting from transient vessel nonperfusion. Blood vessels stained only with H33342 (injected first) are said to have 'closed' during the interval between stain injections. Conversely, vessels outlined only by $DiOC_7(3)$ (injected second) have 'opened'. Use of this terminology does not imply that collapse of the vessel lumen is responsible for staining mismatch, only that the vessel was temporarily *non-perfused*. Double-labelling experiments were performed in s.c. SCCVII tumours and the interval between stain injections was varied from 5 to 60 min. H33342 was injected at a dose of 15 mg $kg^{-1}$ and $DiOC_7(3)$ at a dose of 1 mg $kg^{-1}$. Mice were killed 5 min after $DiOC_7(3)$ injection. In some experiments, fluorescent zinc cadmium sulphide particles were injected in place of $DiOC_7(3)$.

*Radiation response*

To determine if H33342 influenced tumour oxygenation and thus the tumour response to radiation, the stain was administered at varying intervals before and after an X-ray dose of 10 Gy. SCCVII tumours in unanaesthetised, restrained mice were locally irradiated, using parallel opposed fields, at a dose rate of 3.16 Gy $min^{-1}$ using a 250 kVp X-ray source. H33342 was given i.v. in 50 µl volume 20 min before, immediately before, or 20 min after irradiation. Some mice were killed 5 min after H33342 injection and the tumours then irradiated to obtain the response of a completely anoxic cell population. Following treatment, tumours were excised and enzymatically dissociated to a single cell suspension as described previously (Olive *et al.*, 1985). Cells were then incubated for 1–2 min with fluorescein isothiocyanate (FITC)-labelled anti-mouse IgG (whole molecule; Sigma) to stain non-tumour host cells, predominantly macrophages (Olive, 1989). Cells were anlaysed using fluorescence-activated cell sorting (FACS 440, Becton-Dickinson, Sunnyvale, CA, USA): equal numbers of FITC-negative (i.e. tumour) cells were sorted into 10 fractions based on cellular H33342 concentration. The clonogenicity of each fraction was assessed by plating cells, counted by the cell sorter, into 100 mm plastic cell culture plates and incubating in 5% $O_2$, 5% $CO_2$, and 90% $N_2$ at 37°C for 10 days. Colonies were then stained with malachite green and counted.

### Results

*Tumour blood flow*

H33342 caused a dose-dependent reduction in tumour red blood cell (RBC) flow in the SCCVII carcinoma as measured by laser Doppler flowmetry (Figures 1 and 2). In immobilised mice (ketamine/diazepam anaesthesia) bearing subcutaneous back tumours, an H33342 dose of 15 mg $kg^{-1}$ reduced RBC flow to 19 ± 11% of normal with a return to pretreatment flow in <7 min (Figure 1). In foot tumours, the decline in RBC flow was less pronounced in both anaesthetised (50 ± 15% of normal) and unanaesthetised mice (52 ± 14% of normal). In foot tumours, RBC flow was significantly different from pretreatment values for only 2–3 min post-injection. A dose of 5 mg $kg^{-1}$ was not significantly vasoactive (Figure 2). No long-term reductions in flow were observed using this method. In back tumours, the number of moving RBCs (indicative of functional microvascular volume) also declined following H33342 administration (15 mg $kg^{-1}$) (59% of normal, $P < 0.001$) but this effect was much less pronounced in foot tumours (74% and 89% of normal with and without anaesthesia respectively, $P < 0.05$). Most of the tumour blood flow reduction induced by H33342 was a result of a decrease in mean RBC velocity: in back tumours RBC velocity declined to 31% of pretreatment values while in foot tumours velocity decreased to 62% in anaesthetised mice and to 58% in unanaesthetised mice ($P < 0.001$). Blood flow reduction was observed in tumours ranging in size from 50 to 1,000 mg. Flow reduction in large back tumours > 800 mg was not significantly different than that of small tumours < 200 mg (19 ± 12% of pretreatment values *vs* 21 ± 7%). H33342 had no significant effect on blood flow in skin of a non-tumour-bearing foot.

**Figure 1** Effect of H33342 (15 mg $kg^{-1}$ i.v.) on red blood cell (RBC) flow, number of moving RBCs (indicative of functional microvascular volume), and mean RBC velocity as assessed by laser Doppler flowmetry. SCCVII tumours were either implanted subcutaneously over the sacral region and mice immobilised with ketamine/diazepam anaesthesia (■) ($n = 8$) or tumours were implanted subcutaneously in the hindfoot dorsum: (●) anaesthesia ($n = 5$), (○) no anaesthesia ($n = 6$). Error bars represent s.e.m. In back tumours, RBC flow, from 1 to 6 min inclusive, is significantly different ($P < 0.05$) than pretreatment values. In foot tumours, RBC flow is only significantly different ($P < 0.05$) than pretreatment values at 1 and 2 min post-H33342 injection.

**Figure 2** Effect of i.v. H33342 dose on tumour RBC flow. Measurements were made in SCCVII tumours implanted subcutaneously over the sacral region. Mice were immobilised with ketamine/diazepam anaesthesia. ○, Maximum reduction in RBC flow. ●, Duration of flow reduction, i.e. time to return to pretreatment values. Error bars represent s.d.

The tumour blood flow reduction observed following H33342 injection is unlikely to be an artifact of the measurement technique since the absorption spectrum of H33342 does not overlap with that of the infrared laser (780 ± 20 nm) and the stain did not alter the laser Doppler DC voltage signal, i.e. H33342 staining did not affect the amount of light scattered by stationary tumour cells (data not shown).

**Figure 3** Effect of H33342 (15 mg $kg^{-1}$ i.v. in 50 µl injection volume) on mean arterial blood pressure (MABP) (●) and heart rate (HR) (○) in 8-week-old male C3H/He mice ($n = 5$) anaesthetised with halothane via vapouriser. Error bars represent s.d. The maximum decline in blood pressure was 20 ± 6 mmHg (range 11–26 mmHg) which is statistically significant ($P < 0.005$). No significant change in heart rate was observed.

*Blood pressure/heart rate*

H33342 caused a transient reduction in mean arterial blood pressure measured directly in anaesthetised C3H/He mice (Figure 3). Blood pressure declined following i.v. H33342 injection in all five animals tested. Pretreatment blood pressure was 86 ± 4 mmHg. The minimum pressure recorded after H33342 injection was 66 ± 9 mmHg and thus the maximum decrease in blood pressure was 20 ± 6 mmHg (range 11–26 mmHg; $P < 0.005$). Blood pressure remained significantly lower than pretreatment values up to 23 min after H33342 injection ($P < 0.05$), although a return to near baseline levels occurred by 14 min. Heart rate was not significantly changed.

*Metabolic parameters*

H33342 at concentrations as high as 400 µg $ml^{-1}$ had no significant effect on oxygen utilisation by SCCVII cells *in vitro*. A concentration of 400 µg $ml^{-1}$ would be expected to produce tumour intracellular H33342 concentrations at least 100 × those achievable *in vivo* (Olive *et al.*, 1985). Oxygen consumption (relative to untreated cells) was 0.99 ± 0.07 ($n = 6$) and 0.92 ± 0.08 ($n = 10$) for H33342 concentrations of 100 µg $ml^{-1}$ and 400 µg $ml^{-1}$ respectively. Venous blood pH, $pO_2$, and $pCO_2$ were not significantly altered following H33342 administration (Table I). A slight drop in systemic haematocrit was noted but this was also seen in control animals and was likely the result of repeated orbital sinus blood sampling. No effect on the oxygen-haemoglobin dissociation curve was observed. H33342 had no effect on rectal skin, or tumour temperature (data not shown).

*Transient tumour perfusion*

In a previous report (Trotter *et al.*, 1989*a*), when H33342 and $DiOC_7(3)$ injections were separated by a 20 min interval, 8.9 ± 2.4% of vessels in 500 mg s.c. SCCVII tumours exhibited staining mismatch indicative of transient perfusion. Small tumours (⩽ 100 mg) did not show significant levels of staining mismatch.

To further validate the use of H33342 as the first vascular marker in a double staining regimen, the interval between stain injections was varied between 5 and 60 min. If H33342-induced transient reductions in tumour perfusion affect the staining mismatch levels obtained then mismatch should decline with increasing injection interval. In addition, vessel closing would presumably predominate relative to vessel opening. No significant difference in mismatch levels was noted for injection intervals of 15 to 60 min (Table II). For all intervals, the overall mismatch was significantly greater than when stains were injected simultaneously ($P<0.01$). The overall staining mismatch observed using a 20 min interval in this series of experiments (performed as a group over a 2-month period) was 5.4 ± 1.8% ($n = 15$, tumour weight 560 ± 210 mg) a value less than the 8.9 ± 2.4% found in earlier similar experiments (Trotter *et al.*, 1989*a*). Similar tumour transplant generations were used and we have no explanation for the slightly reduced level of staining mismatch observed in the present study.

An injection interval of 5 min resulted in a highly variable, artifactually elevated staining mismatch (11.8 ± 11.7%) with a disproportionate amount of vessel closure (H33342, no $DiOC_7(3)$) (Figure 4) due to the vasoactive effect of H33342. Apart from this 5 min injection interval, no significant increase in vessel closing relative to vessel opening was observed.

If fluorescent particles were used in place of $DiOC_7(3)$ (Chaplin *et al.*, 1987; Jirtle, 1988) then vessels marked with intraluminal particles and not H33342 represented those that opened in the interval between injections. In SCCVII tumours, 6.4% of vessels were found to have opened using this method (data not shown), demonstrating that $DiOC_7(3)$ itself is not responsible for reperfusion of previously nonperfused vessels.

*Radiation response*

The overall radiation response of SCCVII tumour cells depended on the time of H33342 injection relative to radiation treatment. H33342 injection 20 min before or 20 min after irradiation resulted in essentially identical tumour cell survival in all sort fractions (Figure 5). If, however, tumours were irradiated during the period of reduced tumour blood flow following H33342 injection, i.e. administration of H33342 immediately prior to irradiation, the cell survival was elevated compared to the survival observed when H33342 injection and radiation were separated by 20 min although this increase is only statistically significant ($P<0.01$) in

**Table I** Effect of H33342 (15 mg $kg^{-1}$ i.v.) on venous blood gases, pH, oxyhaemoglobin saturation ($HBO_2$), and haematocrit (HCt) in unanaesthetised C3H/He mice

| | *Pretreatment* | *5 minutes* | *20 minutes* |
|---|---|---|---|
| pH | 7.33±0.01 | 7.33±0.01 | 7.27±0.07 |
| $pCO_2$ (mmHg) | 44±3 | 37±2 | 41±3 |
| $pO_2$ (mmHg) | 34±2 | 35±3 | 34±5 |
| $HBO_2$ (%) | 38±3 | 40±5 | 38±10 |
| HCt (%) (no drug) | 46±2 | 42±1 | 40±1 |
| HCt (%) (H33342) | 46±2 | 43±1 | 42±3 |

Parameters were measured prior to, 5 min after, and 20 min after H33342 injection. Means±s.d. for 5–7 mice are shown.

**Table II** Effect of the interval between stain injections (INT) on prefusion mismatch in SCCVII carcinoma implanted subcutaneously in the back

| *INT (min)* | *n* | *Weight (mg)* | *Open (%)* | *Closed (%)* | *Total (%)* |
|---|---|---|---|---|---|
| 0 | 11 | 0.70±0.53 | 0.98±0.37 | 0.34±0.38 | 1.34±0.50 |
| 5 | 8 | 0.50±0.11 | 1.05±0.71 | 10.7±11.9[b] | 11.8±11.7[b] |
| 10 | 7 | 0.51±0.07 | 1.70±0.66[b] | 1.06±0.72[b] | 2.75±1.04[b] |
| 15 | 9 | 0.66±0.11 | 3.43±2.53[b] | 2.28±2.84[a] | 5.72±3.28[c] |
| 20 | 15 | 0.56±0.21 | 2.72±1.39[c] | 2.71±1.97[c] | 5.41±1.77[c] |
| 30 | 8 | 0.70±0.22 | 3.05±1.60[c] | 1.95±1.24[c] | 5.00±1.94[c] |
| 45 | 8 | 0.74±0.23 | 3.20±2.80[a] | 3.26±2.21[c] | 6.48±3.05[c] |
| 60 | 9 | 0.60±0.17 | 1.74±1.35 | 3.33±2.72[b] | 5.04±2.74[c] |

The percentage of total vessels exhibiting stianing mismatch is shown (mean ± s.d.); this value is further subdivided into vessels which 'opened' ($DiOC_7(3)$, no H33342) and those which 'closed' (H33342, no $DiOC_7(3)$) during the interval between stain injections. Levels of statistical significance for non-simultaneous injections *vs* controls (interval = 0) are as follows: [a]$P<0.05$; [b]$P<0.01$; [c]$P<0.001$.

**Figure 4** Effect of the interval between stain injections on vessel 'opening' ( ▨ ) and vessel 'closing' ( ■ ) in SCCVII carcinoma implanted subcutaneously in the back. Values are expressed as a percent (± s.em., $n = 7-15$) of all mismatched vessels. Differences between the amount of vessel opening and the amount of closing are not statistically significant with the exception of interval = 5 min where closing (10.7 ± 11.9%, mean ± s.d.) exceeded opening (1.05 ± 0.71%, mean ± s.d.) ($P<0.025$) due to the transient effects of H33342 on tumour blood flow.

**Figure 5** Effect of H33342 bolus injection (15 mg $kg^{-1}$ i.v.) on the response of subcutaneous SCCVII tumour cells to 10 Gy tumour-localised X-irradiation in restrained, unanaesthetised male C3H/He mice. Control plating efficiency (**a**, mean ± s.d.) is plotted as a function of H33342 sort fraction (based on H33342 concentration); brightly stained cells are those located immediately adjacent to the tumour blood supply. **b**, shows the surviving fraction of tumour cells after 10 Gy X-rays: H33342 was injected 20 min prior to X-rays ($n = 5$) (△); 20 min after X-rays ($n = 5$) (▲); or immediately prior to X-rays ($n = 7$) (●). The responses of full oxic SCCVII tumour cells irradiated *in vitro* (□) and of completely anoxic cells (killed animal; $n = 5$) (○) are also shown for comparison. The average response to radiation in all sort fractions is indicated on the left of the graph by (▷) and (▶). In **b** error bars represent s.e.m.

dimly stained cells distant from the blood supply. These results are consistent with observations that H33342 transiently reduced tumour perfusion; reduction in tumour blood flow will cause impaired oxygen delivery, tumour cell hypoxia, and a relative resistance to X-irradiation.

### Discussion

The results of this study clearly indicate that the vascular marker H33342 has vasoactive properties in the C3H/He mouse; the stain causes a decrease in tumour blood flow and a transient decline in mean arterial blood pressure. Several important observations require emphasis: (1) reductions in tumour RBC flow are dose-dependent and transient; (2) $5\,mg\,kg^{-1}$ H33342 is not significantly vasoactive; (3) flow reductions are independent of tumour size; (4) foot tumours show a smaller, $<3$ min reduction in RBC flow; (5) anaesthesia does not potentiate the maximum H33342-induced flow reduction seen in foot tumours; (6) H33342 has no significant effect on skin RBC flow.

The mechanism responsible for H33342-induced reductions in tumour perfusion is not clear, but, based on observations by Algire and Legallais (1951), we hypothesised that small changes in mouse blood pressure might explain the apparently selective decrease in tumour flow. Indeed, H33342 causes a transient but significant decline in mouse blood pressure measured by direct arterial cannulation in anaesthetised animals. The time course of blood pressure changes is not, however, identical to that of tumour blood flow reductions, and therefore, perturbations in systemic pressure probably do not entirely explain H33342-induced effects on tumour perfusion. A direct effect of H33342 on tumour blood vessels is possible, but no obvious reason for this selectivity is immediately apparent.

A reduction in blood flow results in decreased tumour oxygenation as evidenced by the increased cell survival seen if tumours are irradiated immediately after H33342 injection. This H33342-induced radioresistance is unlikely to be due to a direct radioprotective effect of the stain (Smith & Anderson, 1984; Young & Hill, 1989) since no tumour cell radioprotection has been observed even after *in vivo* administration of $400\,\mu g\,g^{-1}$ (Young & Hill, 1989), a dose $25\times$ that used in the radiobiologic experiments described in this study.

H33342 has no significant influence on other physiologic/metabolic parameters measured. Heart rate, temperature, venous blood gases, pH, haematocrit, and oxyhaemoglobin saturation are unchanged. Exposure of SCCVII tumour cells *in vitro* to high H33342 concentrations has no effect on relative oxygen utilisation rate of the cells.

We believe that H33342-induced reductions in tumour blood flow are not causally related to the phenomena of transient tumour perfusion and acute hypoxia. Several lines of evidence support this conclusion. (1) Mean tumour cell fluorescence increases linearly as a function of injected H33342 dose (Chaplin & Acker, 1987) indicating that large doses of H33342, while vasoactive, do not cause instantaneous tumour vessel non-perfusion and reduced stain delivery. (2) In double-labelling experiments, approximately equal numbers of opening vessels and closing vessels are observed at intervals between stain injections of $>5$ min. If H33342 had prolonged adverse effects on tumour perfusion, vessel closing would be expected to predominate over vessel opening. Vessel opening can also be demonstrated if fluorescent particles are used in place of $DiOC_7(3)$ as the second vascular marker. An ideal control experiment for the double staining method would involve injection of $DiOC_7(3)$ at some interval *before* H33342, i.e. the sequence of stain injection would be reversed. However, $DiOC_7(3)$, like H33342, causes an abrupt transient decrease in tumour RBC flow, but this is followed, after a brief period of recovery, by a significant prolonged decline in perfusion (Trotter *et al.*, 1989*b*). This effect precludes the use of $DiOC_7(3)$ as a first marker in a double staining regimen. (3) Small SCCVII tumours do not exhibit staining mismatch indicative of transient vessel nonperfusion (Trotter *et al.*, 1989*a*) and yet such tumours show a flow reduction after H33342 identical to that observed in larger tumours. Thus, the degree of H33342-induced reduction in tumour blood flow is not causally linked to transient vessel nonperfusion. (4) H33342 injection 20 min prior to X-irradiation results in the same tumour cell survival as when the stain is injected 20 min *after* irradiation. Therefore, in support of the results obtained with laser Doppler flowmetry, H33342 reductions in tumour oxygenation are short-lived and no evidence of radiobiologic hypoxia is observed 20 min after stain injection.

These results indicate that H33342 can be used as a marker of functional tumour vasculature in experiments designed to identify transient perfusion (or the resulting acute hypoxia) provided several limitations are kept in mind. First, high doses of H33342 should be avoided. Unfortunately, the non-vasoactive dose (in anaesthetised mice) of $5\,mg\,kg^{-1}$ does not provide sufficient fluorescence in tumour tissue sections to allow accurate vessel counting using fluorescence microscopy. A slightly higher dose might perhaps be employed when using tumours grown in other sites (e.g. foot) and when anaesthesia is avoided. In our laboratory, cell sorting experiments are routinely performed using *in vivo* H33342 doses of $10\,mg\,kg^{-1}$ administered to *un*anaesthetised mice. Second, because H33342 reduces tumour perfusion for several minutes, the second marker in a double staining protocol should be injected at least 7 min after H33342, when (following doses $\leqslant 15\,mg\,kg^{-1}$) flow in back tumours has returned to pretreatment levels. This places a limit on the temporal resolution achievable using this technique. Similarly, H33342 should be injected at least 7 min before irradiation, or alternatively, after irradiation. It should be stressed that these conclusions apply only to the SCCVII carcinoma implanted subcutaneously in the back. Shorter intervals can likely be safely employed using foot tumours. Other tumours types or implantation sites may exhibit a different response to H33342 injection. It should be noted that in SCCVII foot tumours, laser Doppler measurements were performed with a surface probe; sampling of some skin microvasculature may explain the less pronounced RBC flow reduction induced by H33342 in this tumours site compared to back tumours (probe placed in direct contact with peripheral tumour tissue). Finally, the effect of intravenous *infusion* of H33342 was not examined in this study; preliminary results suggest that infusion of $10\,mg\,kg^{-1}$ H33342 (dose required for cell sorting) over 20–30 min causes no changes in SCCVII RBC flow (unpublished observations).

In summary, H33342 has vasoactive properties; that is, the stain causes a decrease in mean arterial blood pressure and a transient dose-dependent decline in tumour blood flow. These effects impose certain temporal restrictions when the stain is employed in histological and radiobiological techniques. If these limitations are observed, the use of H33342 does not appear to be causally related to the phenomena of transient perfusion and acute hypoxia.

The authors are indebted to C. Toth, C. Vikse, B. Pearson, C. Peter and I.-M. Johansen for technical assistance. This work was supported by USPHS grant CA-37879. Martin Trotter is the recipient of a Medical Research Council (Canada) Research Fellowship.

### References

ALGIRE, G.H. & LEGALLAIS, F.Y. (1951). Vascular reactions of normal and malignant tissues *in vivo*. IV. The effect of peripheral hypotension on transplanted tumors. *J. Natl Cancer Inst.*, **12**, 399.

ARNDT-JOVIN, D.J. & JOVIN, T.M. (1977). Analysis and sorting of living cells according to deoxyribonucleic acid content. *J. Histochem. Cytochem.*, **25**, 585.

BIAGLOW, J.E. & DURAND, R.E. (1976). The effects of nitrobenzene derivatives on oxygen utilization and radiation response of an *in vitro* tumor model. *Radiat. Res.*, **65**, 529.

CHAPLIN, D.J. & ACKER, B. (1987). The effect of hydralazine on the tumor cytotoxicity of the hypoxic cell cytotoxin RSU-1069: evidence for therapeutic gain. *Int. J. Radiat. Oncol. Biol. Phys.*, **13**, 579.

CHAPLIN, D.J., DURAND, R.E. & OLIVE, P.L. (1985). Cell selection from a murine tumour using the florescent perfusion probe Hoechst 33342. *Br. J. Cancer*, **51**, 569.

CHAPLIN, D.J., DURAND, R.E. & OLIVE, P.L. (1986). Acute hypoxia in tumors: implications for modifiers of radiation effects. *Int. J. Radiat. Oncol. Biol. Phys.*, **12**, 1279.

CHAPLIN, D.J., OLIVE, P.L. & DURAND, R.E. (1987). Intermittent blood flow in a murine tumor: radiobiological effects. *Cancer Res.*, **47**, 597.

DURAND, R.E. (1982). Use of Hoechst 33342 for cell selection from multicell systems. *J. Histochem. Cytochem.*, **30**, 117.

DURAND, R.E., CHAPLIN, D.J. & OLIVE, P.L. (1990). Cell sorting with Hoechst or carbocyanine dyes as perfusion probes in spheroids or tumors. In *Methods in Cell Biology, Volume 33: Flow Cytometry*, Crissman, H. & Darzynkiewicz, Z. (eds). Academic Press: Orlando.

DURAND, R.E. & OLIVE, P.L. (1982). Cytotoxicity, mutagenicity, and DNA damage by Hoechst 33342. *J. Histochem. Cytochem.*, **30**, 111.

FALLOWFIELD, M.E. (1989). Vascular volume in B16 allografts and human melanoma xenografts estimated by means of Hoechst 33342. *J. Pathol.*, **157**, 249.

HAUMSCHILD, D.J. (1986). Microvascular blood flow measurement by laser-Doppler flowmetry. *TSI Application Note*. St Paul, MN.

JIRTLE, R.L. (1988). Chemical modification of tumour blood flow. *Int. J. Hypertherm.*, **4**, 355.

LOEFFLER, D.A., KENG, P.C., WILSON, K.M. & LORD, E.M. (1987). Comparison of fluorescence intensity of Hoechst 33342-stained EMT6 tumour cells and tumour-infiltrating host cells. *Br. J. Cancer*, **56**, 571.

MINCHINTON, A.I., DURAND, R.E. & CHAPLIN, D.J. (1990). Intermittent bloodflow in the KHT sarcoma – flow cytometry studies using Hoechst 33342. *Br. J. Cancer*, **62**, 195.

MURRAY, J.C., RANDHAWA, V. & DENEKAMP, J. (1987). The effects of melphalan and misonidazole on the vasculature of a murine sarcoma. *Br. J. Cancer*, **55**, 233.

OLIVE, P.L. (1989). Distribution, oxygenation, and clonogenicity of macrophages in a murine tumor. *Cancer Comm.*, **1**, 93.

OLIVE, P.L., CHAPLIN, D.J. & DURAND, R.E. (1985). Pharmacokinetics, binding and distribution of Hoechst 33342 in spheriods and murine tumours. *Br. J. Cancer*, **52**, 739.

OLIVE, P.L. & DURAND, R.E. (1987). Characterization of a carbocyanine derivative as a fluorescent penetration probe. *Cytometry*, **8**, 571.

REINHOLD, H.S. & VISSER, J.W.M. (1983). *In vivo* fluorescence of endothethial cell nuclei stained with the bis-benzamide H33342. *Int. J. Microcirc. Clin. Exp.*, **2**, 143.

SHEPHERD, A.P., RIEDEL, G.L., KIEL, J.W., HAUMSCHILD, D.J. & MAXWELL, L.C. (1987). Evaluation of an infrared laser-Doppler blood flowmeter. *Am. J. Physiol.*, **252** (*Gastrointest. Liver Physiol.*, **15**), G832.

SIEMANN, D.W. & KENG, P.C. (1988). Characterization of radiation resistant hypoxic cell subpopulations in KHT sarcomas. (ii) Cell sorting. *Br. J. Cancer*, **58**, 296.

SMITH, K.A., HILL, S.A., BEGG, A.C. & DENEKAMP, J. (1988). Validation of the fluorescent dye Hoechst 33342 as a vascular space marker in tumours. *Br. J. Cancer*, **57**, 247.

SMITH, P.J. & ANDERSON, C.O. (1984). Modification of the radiation sensitivity of human tumor cells by a bis-benzimidazole derivative. *Int. J. Radiat. Biol.*, **46**, 331.

TROTTER, M.J., CHAPLIN, D.J., DURAND, R.E. & OLIVE, P.L. (1989*a*). The use of fluorescent probes to identify regions of transient perfusion in murine tumors. *Int. J. Radiat. Oncol. Biol. Phys.*, **16**, 931.

TROTTER, M.J., CHAPLIN, D.J. & OLIVE, P.L. (1989*b*). Use of a carbocyanine dye as a marker of functional vasculature in murine tumours. *Br. J. Cancer*, **59**, 706.

TROTTER, M.J., ACKER, B.D. & CHAPLIN, D.J. (1989*c*). Histological evidence for nonperfused vasculature in a murine tumor following hydralazine administration. *Int. J. Radiat. Oncol. Biol. Phys.*, **17**, 785.

YOUNG, S.D. & HILL, R.P. (1989). Radiation sensitivity of tumour cells stained *in vitro* or *in vivo* with the bisbenzimide fluorochrome Hoechst 33342. *Br. J. Cancer*, **60**, 715.

ZWI, L.J., BAGULEY, B.C., GAVIN, J.B. & WILSON, W.R. (1989). Blood flow failure as a major determinant in the antitumour action of flavone acetic acid. *J. Natl Cancer Inst.*, **81**, 1005.